# Korean Institute of Medical Education and Evaluation Presidential Address: the role of KIMEE as a medical education accreditation agency during the coronavirus disease 2019 pandemic

**DOI:** 10.3352/jeehp.2021.18.2

**Published:** 2021-02-23

**Authors:** Young Chang Kim

**Affiliations:** President, Korean Institute of Medical Education and Evaluation, Seoul, Korea; Hallym University, Korea


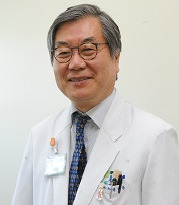


(photo of Dr. Young Chang Kim)

The new year of 2021 arrived amidst the coronavirus disease 2019 (COVID-19) pandemic, which changed many patterns of daily life, to the point that the current era is sometimes referred to as the “new normal.” Education for health professionals is no exception to this trend, as many classroom lectures were changed to non-face-to-face online classes. It has been challenging for medical schools to prepare a new online education system in the early stages of the COVID-19 pandemic, especially because bedside clinical clerkships require meeting patients face-to-face in the hospital or clinic. To achieve the objectives of clinical education, some medical schools have adopted simulation programs whenever possible, while other schools have continued bedside teaching with appropriate protection procedures for students. A silver lining of the COVID-19 pandemic may be that it has provided an opportunity to promote the digital transformation of medical education.

## Accreditation in 2020

To cope with this situation, the Korean Institute of Medical Education and Evaluation (KIMEE) announced on July 3, 2020 that all online education from March 2020 to February 2021 would be recognized as fulfilling educational accreditation requirements. The conditions for fulfillment included confirmation of participating in online education and the provision of information on the precise methods used to evaluate students’ achievement. According to this policy, educational institutions have provided enough evidence that an equivalent educational effect has been achieved through online education.

KIMEE took several important measures during the COVID-19 pandemic in 2020. First, KIMEE set new guidelines for the accreditation process, prioritizing safety and containment measures, and we also prepared several scenarios to apply the guidelines according to the severity of the pandemic. Initially, the 2 existing most important accreditation procedures (document-based evaluation and site-visit evaluation) were maintained. However, in light of the need to minimize face-to-face contact, the number of evaluation teams and the duration of site visits were reduced to the lowest level that would not compromise the equivalence of accreditation. Instead, the document-based evaluation process was strengthened so that at least 2 meetings were held, and efforts were made to minimize the need for confirmation through site visits to the greatest possible extent. The document-based evaluations were done online and offline, but the site-visit evaluations were carried out face-to-face with protective measures as a top priority.

KIMEE disclosed the accreditation results for 12 medical schools in 2020 on January 14, 2021. The application by the 12 medical schools was completed on February 29, 2020. After receiving each school’s self-evaluation report as of August 31, 2020, the evaluation team visited each school from October 14 to November 18, 2020. The results were as follows:

The schools that received a 6-year accreditation (March 31, 2021 to February 28, 2027) were Ewha Womans University, Inje University, JeonBuk National University, and Korea University. The schools that received a 4-year accreditation (March 31, 2021 to February 28, 2025) were Catholic Kwandong University, Dongguk University, Hanyang University, Kyung Hee University, Pusan National University, Seoul National University, and Sungkyunkwan University. The Catholic University of Korea also received a 4-year accreditation. However, it was under re-evaluation according to the request of this institute.

A regular 2-year interim evaluation was also done for 11 medical schools in 2020 after receiving a self-interim evaluation report as of August 31, 2020. All subject schools maintained their accreditation, including Cha University, Chonnam University, Chung-Ang University, Dong-A University, Eulji University, Gyeongsang National University, Hallym University, Inha University, Kangwon National University, Konkuk University, and Wonkwang University.

## Accreditation process in 2021

This year’s accreditation criteria also will follow the Accreditation Standards of KIMEE 2019 (ASK 2019) [1], which was prepared as an accreditation standard for medical schools in Korea based on the “WFME Global Standards for Quality Improvement 2015–BME” [2]. It consists of 9 areas, 92 basic criteria, and 51 excellence criteria (Table 1). The ASK 2019 has been used for the accreditation process since 2019. This year is the 3rd year of adopting this new system. It is expected that the accreditation process will also be challenging this year because there have been many changes in the environment of medical education since last year. To overcome these difficulties, in the near future, KIMEE will temporarily adapt the accreditation guideline for application during the COVID-19 pandemic period.

## Congratulations to KIMEE founding members for receiving the KAMS Honor in Medicine Award

On January 12, 2021, 2 previous members of KIMEE, Drs. Chong Wook Lee and Moo Sang Lee, received the 7th KAMS Honor in Medicine Award (*Uihak-gong-heon-sang*) from the Korean Academy of Medical Science (KAMS), which was supported by the Buechepyo GHASONG Foundation, of which the chair of the board is Dr. Doh Joon Yoon. The KAMS Honor in Medicine Award recognizes distinguished physicians who have contributed to medical development in Korea. KIMEE recommended 2 persons as candidates for this award.

**Fig. 1.**
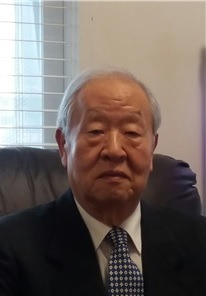
Photo of Dr. Chong Wook Lee (provided by Dr. Chong Wook Lee).

**Fig. 2.**
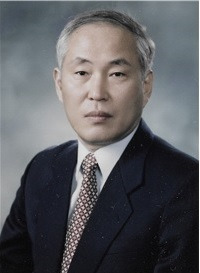
Photo of Dr. Moo Sang Lee (provided by Dr. Moo Sang Lee).

Dr. Chong Wook Lee, a former professor of urology at Seoul National University College of Medicine, was the 1st president of the KIMEE from March 2004 to March 2007 (Fig. 1). Dr. Moo Sang Lee, a former professor of urology at Yonsei University College of Medicine, was the 2nd president of the KIMEE from March 2007 to February 2010 (Fig. 2). It is a great honor for KIMEE to hear the news that these 2 previous contributors to KIMEE received such an outstanding award. Without those pioneers’ sacrifice and voluntary devotion, KIMEE would not have established its present role as an accreditation agency. The members of KIMEE will not forget their endeavors to promote the quality of medical education in Korea.

## KIMEE’s role in supporting other accreditation agencies

KIMEE, as a WFME-recognized accrediting body, has a responsibility to promote other accreditation agencies, both domestically and abroad, based on the experience gained through 20 years of engagement in the accreditation process. There are accreditation agencies in different fields in Korea, including dental education, nursing education, veterinary education, and oriental medicine (*hanuihak*) education. KIMEE has provided guidelines and processes to all other agencies upon request.

KIMEE was asked by the Global Medical Education Development Working Group of the Yonsei Institute for Global Health to provide advice on the establishment of Ho Chi Minh University’s medical curriculum and Vietnamese medical education accreditation agency. In this regard, a meeting with the Working Group was held on August 24, 2020, at KIMEE’s office in Seoul, to discuss management and cooperation plans. An official advisory review was then requested by the Working Group. For the successful implementation of this 5-year project starting in 2021, an advisory committee from KIMEE will be organized, and committee members will be dispatched to Vietnam.

## KIMEE as a co-sponsoring agency of JEEHP

KIMEE became a co-sponsoring agency of the *Journal of Educational Evaluation for Health Professions* (JEEPH) on June 17, 2020 [3]. JEEHP will be a window to the world for KIMEE’s research work. Therefore, it is an excellent opportunity for KIMEE to disseminate information about its activities to numerous medical education accreditation agencies and medical schools worldwide.

The COVID-19 pandemic has brought remarkable changes to basic medical education. There is a saying that every crisis is an opportunity; in that spirit, KIMEE will strive to carry out accreditation and to ensure rigorous medical education this year.

## Figures and Tables

**Table 1.** Accreditation standards of KIMEE 2019 (ASK2019)―Basic Medical Education revised on February 2, 2020

Evaluation area	Sub-area	Revision standard		
		Basic standard	High quality standard	Total
1. Mission and outcomes	1.1 Mission	3	1	4
	1.2 Institutional autonomy and academic freedom	1	-	1
	1.3. Educational outcomes	3	1	4
	1.4. Participation in formulation of mission and outcomes	1	1	2
	Subtotal	8	3	11
2. Curriculum	2.1. Curriculum	3	1	4
	2.2. Scientific method	3	-	3
	2.3. Basic medical sciences	2	1	3
	2.4. Medical humanities	1	1	2
	2.5. Clinical sciences and skills	4	3	7
	2.6. Program structure, composition, and duration	2	2	4
	2.7. Curriculum management	2	-	2
	2.8. Linkage with medical practice and the health sector	1	1	2
	Subtotal	18	9	27
3. Student assessment	3.1. Assessment methods	4	1	5
	3.2. Relation between assessment and learning	4	2	6
	Subtotal	8	3	11
4. Student	4.1. Admission policy and selection	1	3	4
	4.2. Student intake	1	-	1
	4.3. Student counseling and support	6	3	9
	4.4. Student representation	2	-	2
	Subtotal	10	6	16
5. Faculty	5.1. Recruitment and selection policy	6	1	7
	5.2. Faculty activity and development	6	1	7
	Subtotal	12	2	14
6. Education resources	6.1 Physical facilities	8	1	9
	6.2. Clinical training resources	3	1	4
	6.3. Information technology	1	2	3
	6.4. Medical research and fostering medical scientists	3	1	4
	6.5. Educational expertise	2	3	5
	6.6. Educational exchanges	1	1	2
	Subtotal	18	9	27
7. Education evaluation	7.1. Mechanisms for education monitoring and evaluation	3	1	4
	7.2. Teacher and student feedback	1	1	2
	7.3. Performance of students and graduates	1	1	2
	7.4. Involvement of stakeholders	1	-	1
	Subtotal	6	3	9
8. Operation system and administration	8.1. Operation system	4	2	6
	8.2. Academic leadership	1	1	2
	8.3. Educational budget and resource allocation	2	-	2
	8.4. Administrative staff and management	1	1	2
	8.5. Interaction with health sector	1	1	2
	Subtotal	9	5	14
9. Continuous improvement	9.0. Continuous improvement	3	11	14
	Subtotal	3	11	14
Total	36	92	51	143

KIMEE, Korean Institute of Medical Education and Evaluation.
